# Medicine and Missionaries

**Published:** 1895-06-29

**Authors:** 


### Social Problems.

### MEDICINE AND MISSIONARIES.

Although medical missions may be said to date from the sending forth of the Seventy, with the threefold injunction to carry no purse, to heal the sick, and to preach the Gospel, no real union was ever planned till quite recent years between the kindred professions which aim at healing the body and the soul, nor any attempt to unite in the same person the qualifications of the surgeon and the divine.

An interesting account of the rise of this remarkable movement is given in the Memoirs* recently published of W. Burns Thompson. The Edinburgh Medical Missionary Society was first in the field, and its early ventures at home on the Cowgate, and abroad, are described by Dr. Burns Thompson in a spirited and humorous narrative of personal recollections, slightly marred, it is true, by the sectarian phraseology of a bygone day. Dr. Thompson describes himself as converted to the cause in the course of an unsuccessful attempt to visit in one of the worst parts of Edinburgh, by the discovery of the magical effects of a pennyworth of castor oil in winning him the friendship of an Irish virago. This set him upon considering the close connection in the Gospels between healing and teaching, and resulted in the establishment at the famous No. 39, Cowgate, of a training college, in connection with the society's dispensary, for the assistance of students preparing for medical missionary work.

From the very first the Edinburgh Society kept firmly in view the principle that their students should be first-rate doctors. Many have been the leading students of their day and passed with high honours. All have been thoroughly qualified practitioners. It has been fully recognised, to give the words of Sir Thomas Grainger Stewart, "that there was no use whatever in a man going out to the mission fields merely armed with a box of drugs and some trifling knowledge. Now and then he might make a hit, but that would rarely last." And side by side with the scientific spirit, true missionary zeal has always been held a *sine quâ non.* "The ideal medical missionary must be pre-eminent for piety and professional skill." The right adjustment of the proportions in this delicate alliance, so that neither the preacher nor the scientist element may unduly prevail, presents certain difficulties, more obvious perhaps on paper and in theory than in actual practice. The medical missionary is essentially a pioneer. He is enabled by his professional skill to penetrate to regions where the banner of the Gospel, unsupported by gifts of healing, would prove the signal for massacre. The medical station, always intimately associated with the highest Christian endeavour, becomes primarily a centre for the diffusion of medical training—hospitals and dispensaries are founded, native doctors and nurses are trained and sent forth in the faith of Christ, while mission churches and schools, supported by native effort, testify to the reality of the new religious convictions. All this is well illustrated by the history of the work in Madagascar alone. Beginning in 1862, under Dr. Davidson, with a dispensary for out-patients, the work developed with incredible rapidity. From the first, five to six thousand patients were dealt with yearly, almost single-handed. Very shortly the sympathy and goodwill of the Queen of that country were secured, and a hospital was opened under her patronage at Analeky. A college for training medical missionaries and native doctors was next opened, and in 1874 a beginning was made in training missionary nurses in midwifery and general nursing. Those were not days when any high degree of knowledge was expected from the nurse; her chief qualifications were tersely summed up by Dr. Burns Thompson as "piety and gumption." Still, some measure of hospital training was afforded them, which was soon extended to a limited number of native Christian women; and the value of their work may be estimated from the fact that wherever they have gone a school has sprung up originating entirely from the desire of the people for further instruction, and supported by their voluntary contributions.

Almost as soon as a medical education was thrown open to women, female medical missionaries have taken an important part in the movement. And in the East the need for women workers is almost more urgent than for men.

Difficult as it might seem to unite in one person qualifications so diverse, over two hundred medical men, with British degrees or diplomas, are now holding missionary appointments among the heathen, Mohammedan, or Jewish races. Regarded in comparison with the boundless field of missionary enterprise, doubtless the number seems small indeed, and much sympathy must be felt for the efforts in progress on the other side of the Atlantic, under Dr. Dowkonnt, to found a fully-equipped medical missionary college, by means of which the number of workers may be largely augmented. An appeal for funds for this purpose in the form of a pamphlet, bearing the title "Murdered Millions,"* has been widely circulated in this country. It is, unhappily, disfigured by the gross error, only too common in charitable appeals, of detailing horrors for the purpose of rousing a sensation, a line of action which, did the offenders but know it, more often than not results in landing the unpleasing publication in the fire. Apart from this error of taste, it is not clear that work which depends, as has been said, on exceptional combination and adjustment of qualities, will admit of any very sudden extension through the medium of money endowments, liable to attract unsuitable persons by the prospect of free training. This work, of all others, must be built on the basis of a true vocation, must be tested by many years of the painful days of small things, through good report and evil report, finally must be supported by an ever-widening measure of public sympathy, of the sort which judges calmly and gives liberally out of a full conviction, even when difficulties arise and failure threatens, and when the momentary fervour of disgust stirred by the perusal of atrocities has long faded into oblivion.

* "Reminiscences of Medical Missionary Work." By W. Burns Thompson, F.R.C.S.E., F.R.S.E. (Hodder and Stoughton.)

* "Murdered Millions, by George D. Dowkonnt, M.D. (London: Morgan and Scott.)

